# A transfer learning approach for multiclass classification of Alzheimer's disease using MRI images

**DOI:** 10.3389/fnins.2022.1050777

**Published:** 2023-01-09

**Authors:** Rizwan Khan, Saeed Akbar, Atif Mehmood, Farah Shahid, Khushboo Munir, Naveed Ilyas, M. Asif, Zhonglong Zheng

**Affiliations:** ^1^Department of Computer Science and Mathematics, Zhejiang Normal University, Jinhua, China; ^2^School of Computer Science, Huazhong University of Science and Technology, Wuhan, China; ^3^Division of Biomedical Imaging, Department of Biomedical Engineering and Health Systems, KTH Royal Institute of Technology, Stockholm, Sweden; ^4^Department of Computer Science, National University of Modern Languages, Islamabad, Pakistan; ^5^Department of Computer Science, University of Agriculture, Sub Campus Burewala-Vehari, Faisalabad, Pakistan; ^6^Department of Radiology and Diagnostic Imaging, University of Alberta, Edmonton, AB, Canada; ^7^Department of Physics, Khalifa University of Science and Technology, Abu Dhabi, United Arab Emirates; ^8^Department of Radiology, Emory Brain Health Center-Neurosurgery, School of Medicine, Emory University, Atlanta, GA, United States; ^9^Key Laboratory of Intelligent Education Technology and Application of Zhejiang Province, Zhejiang Normal University, Jinhua, China

**Keywords:** Alzheimer's disease, multiclass classification, deep learning, MRI, early diagnosis of AD

## Abstract

Alzheimer's is an acute degenerative disease affecting the elderly population all over the world. The detection of disease at an early stage in the absence of a large-scale annotated dataset is crucial to the clinical treatment for the prevention and early detection of Alzheimer's disease (AD). In this study, we propose a transfer learning base approach to classify various stages of AD. The proposed model can distinguish between normal control (NC), early mild cognitive impairment (EMCI), late mild cognitive impairment (LMCI), and AD. In this regard, we apply tissue segmentation to extract the gray matter from the MRI scans obtained from the Alzheimer's Disease National Initiative (ADNI) database. We utilize this gray matter to tune the pre-trained VGG architecture while freezing the features of the ImageNet database. It is achieved through the addition of a layer with step-wise freezing of the existing blocks in the network. It not only assists transfer learning but also contributes to learning new features efficiently. Extensive experiments are conducted and results demonstrate the superiority of the proposed approach.

## 1. Introduction

Alzheimer's is one of the most crucial causes of dementia all over the world. Several neurological illnesses, including dementia, afflict a sizable portion of the global population. Patients with Alzheimer show more clear symptoms after the age of 60. However, in some cases, as a result of some gene abnormalities, the symptoms may start to show up at a young age (30–50). Alzheimer's gives rise to functional and structural changes in the brain (Hampel et al., 2021). The progression of Alzheimer's disease (AD) from normal control (NC) spans over a number of years with some intermediate stages ranging from the development of early mild cognitive impairment (EMCI) to late mild cognitive impairment (LMCI). These changes can be observed through MRI images and blood plasma spectroscopy (Pan et al., 2020; Palmer et al., 2021).

Visualizing the MRI scans can somehow allow the physicians to detect the contraction of the gray matter. However, it is a complex process to detect these changes manually. Machine learning-based techniques for the classifications, such as support vector machines (SVMs), artificial neural networks (ANNs), and deep learning-based convolutional neural networks (CNNs), remained very useful in the detection of these minor tissue-level changes (Mehmood et al., 2021). It is important to note that SVM and ANN give local and global optimization-based solutions. However, deep learning-based CNNs consider the feature extraction and learning in the model itself and are considered to be more useful in medical image analysis (Pan et al., 2020; Cheng et al., 2022). But these methods are data-hungry and demand large-scale training datasets to learn the task (i.e., classification in this case) from scratch (Chen et al., 2022; Khan et al., 2022).

Recent advancements in imaging technology, including computerized axial tomography (CT), magnetic resonance images (MRI), and positron emission tomography (PET) (Masdeu et al., 2005; Han et al., 2022) images, have revolutionized the detection of Alzheimer's. Due to ionization effects and cost and computational complexity, gathering a large-scale data set for a particular task is very challenging. The 3D MRI images produced through high-dimensional diagnostic equipment contain several images in a single voxel. These voxels can help to diagnose Alzheimer's at an early stage (Zhang et al., 2019).

A deep Siamese convolution neural network (SCNN) for the multiclass classification of AD is proposed by Mehmood et al. (2020). A natural image-based network to represent neuroimaging data (NIBR-Net) is another significant approach in the target domain, based on a sparse autoencoder (Gupta et al., 2013), where the network learns from a set of bases from natural images with the help of convolution to extract features from the Alzheimer's Disease Neuroimaging Initiative (ADNI) dataset. This method selects the useful features in a single hierarchy while iteratively filtering the undesirable features. Considering the multiple classes of Alzheimer a deep learning-based multiclass classifier is proposed by Farooq et al. (2017). Apart from this, several computer-aided techniques have been suggested for diagnosing AD, especially in the case of severe dementia (Petot and Friedland, 2004; Acharya et al., 2021). Similarly, the issue of the imbalance of class data is handled by Murugan et al. (2021) with the help of a deep DEMNET by using the pre-processed dataset.

Training a neural network with a small-scale dataset (i.e., MRIs) with a higher prediction rate and a higher accuracy is a great challenge. In this study, we propose to utilize the pre-trained VGG16 and VGG19 models to predict NC, EMCI, LMCI, and AD. We extract the gray matter (GM) from the brain MRIs because using entire voxels or raw data directly to train neural networks also presents data management. We applied skull striping and tissue segmentation operations to segregate the full brain MRIs. Considering the data-hungry nature of the neural networks, we proposed to apply data augmentation to the extracted GM slices. It also resolves the challenges of overfitting, which is often aroused due to the unavailability of large-scale dataset. In addition, we step-wise freeze the blocks and add layers to transfer the features for accurate predictions on four classes of the input data, i.e., NC, EMCI, LMCI, and AD. Thus, we resolve the challenges of prospective fluctuations and imbalanced data size and class variation problems. The overall experimental evaluations and comparison with several state-of-the-art approaches demonstrate that the proposed transfer learning method outperformed the extant techniques, making it more suitable for future interactive Alzheimer's applications.

## 2. Related work

The fundamental causes of Alzheimer's are still unknown, and it is believed to be genetic (Adami et al., 2018). Alzheimer's affects a number of social cognitive capacities and results in several neurological conditions that are memory-related (Ramzan et al., 2020). According to an estimate, by the year 2050, 131.5 million individuals will be globally affected by Alzheimer's disease (AD) (Prince et al., 2015). It will become the top cause of death for older people as the number of patients grows daily. High-dimensional data from imaging modalities like MRI, fMRI, PET, amyloid-PET, diffusion tensor imaging, and neurological tests are essential parts of the existing strategies for the diagnosis (Tanveer et al., 2020; Afzal et al., 2021). However, differentiating between the patterns with radiological readings is still quite a difficult task due to the complexity of the minute patterns. As a result, it is difficult to establish an early diagnosis of Alzheimer's. Recently, several deep learning and machine learning-based techniques to enhance picture quality have been presented (Khan et al., 2019; Alenezi and Santosh, 2021). Additionally, the feature extraction- and classification-based approaches can be regularly employed to create prediction models for applications based on intelligent and expert systems (Mehmood et al., 2021; Shams et al., 2021; El-Hasnony et al., 2022).

The progression of Alzheimer's took several years, ranging from NC to MCI and AD. The development of MCI is also considered as early and late MCI (Sperling et al., 2011; Huang et al., 2018). It is imperative to diagnose the disease at an early stage, which is only possible through an accurate classification of various stages of disease (Khan et al., 2022). The recent developments in machine learning and deep learning-based methods can significantly contribute to the target domain (Tanveer et al., 2020). The extraction of the feature and identification of these features based on these techniques can ease the burden on the healthcare system (Razzak et al., 2022). The main objective of the binary class and multi-class classification is to distinguish the features of normal images from impaired images to detect the stage of the disease. Support vector machines (SVM), K-nearest neighbors (KNNs), fuzzy learning, decision trees, random forests, and dimensionality reduction algorithms, like principal component analysis, are readily used methods in traditional machine learning research (Vecchio et al., 2020). The feature extraction through CNNs and deployment of CNNs have revolutionized the whole process (Bi et al., 2020; Liang and Gu, 2021) and can yield acceptable results for the early detection of AD. CNN-based methods can adeptly learn the input images' features to identify a particular disease stage. Hao et al. (2020) suggested a multi-modal framework to extract neurological information from the MRIs in order to classify the various phases of dementia. In order to classify AD, NC, and MCI, Tong et al. developed a multimodal classification framework based on MRI, FDG, and PET scans (Tong et al., 2017). Other recent approaches for the classification of Alzheimer's (Tajbakhsh et al., 2016; Jain et al., 2019; Khan et al., 2019, 2022) can somehow resolve the challenges and some other tries to introduce some transfer learning based approaches (Afzal et al., 2021; Mehmood et al., 2021). However, it is still challenging to extract accurate features to distinguish the images and diagnose the disease at an early stage.

Considering the MRIs, a multi-modal framework is proposed to extract neurological features for the classification of various stages of dementia (Hao et al., 2020). Tong et al. proposed a multimodal classification for AD based on MRI, FDG, and PET images to classify AD, NC, and MCI. A multi-modal learning-based network (Liu et al., 2014) based on PET, and MRI images, a multi-modal stack-net (Shi et al., 2017), and a similarity matrix-based method are proposed by Zhu et al. (2014) based PET, CSF, and MRI biomarkers to distinguish various stages of AD. Some methods rely on the extraction of 2D slices, and some others utilize the whole voxel to distinguish several categories of disease (Payan and Montana, 2015; Islam and Zhang, 2018). A 3D deeply supervised adaptable convolutional neural network (CNN-3D) is proposed by Hosseini-Asl et al. (2018) to predict AD without relying on skull striping with generic feature learning through bio-markers. However, the data management challenges are still complex when handling medical images. In the case of the target problem gathering, a large-scale dataset is a great challenge as compared to ordinary computer vision and image processing tasks as we know that deep learning-based models are data-hungry and demand a large-scale dataset. Therefore, transfer learning approaches (Aderghal et al., 2018; Li et al., 2018; Basaia et al., 2019) are preferred, which utilize the weights from the pretrained models on the large scale datasets such as ImageNet (Mehmood et al., 2021).

To categorize the various stages of the disease with transfer learning, a deep Siamese convolution neural network (SCNN) for the multiclass classification of AD is proposed by Mehmood et al. (2020). A natural image-based network to represent neuroimaging data (NIBR-Net) is another significant approach in the target domain, based on sparse autoencoder (Gupta et al., 2013), where the network learns from a set of bases from natural images with the help of convolution to extract features from the ADNI dataset. A sparse multi-tasking deep learning-based method is proposed by Suk et al. (2016) with a feature adaptive weighting system. This method selects the useful features in a single hierarchy while iteratively filtering the undesirable features. Considering the multiple classes of Alzheimer, a deep learning-based multiclass classifier is proposed by Farooq et al. (2017). Apart from this, several computer-aided techniques have been suggested for diagnosing AD, especially in the case of severe dementia (Petot and Friedland, 2004). These models can assist the physicians in combination with computer-aided intelligent and expert systems (Jo et al., 2019) In this article, we proposed a multi-class classification network by using transfer learning through VGG architecture. We applied a step-wise block freezing strategy to the VGG-16 and VGG-19 models with some additional layers. The proposed method achieves higher accuracy and is capable of working with a small-scale dataset. The overall experimental evaluations demonstrate the superiority of the proposed method as compared to the state-of-the-art approaches.

## 3. The proposed methodology

**Figure 1:**
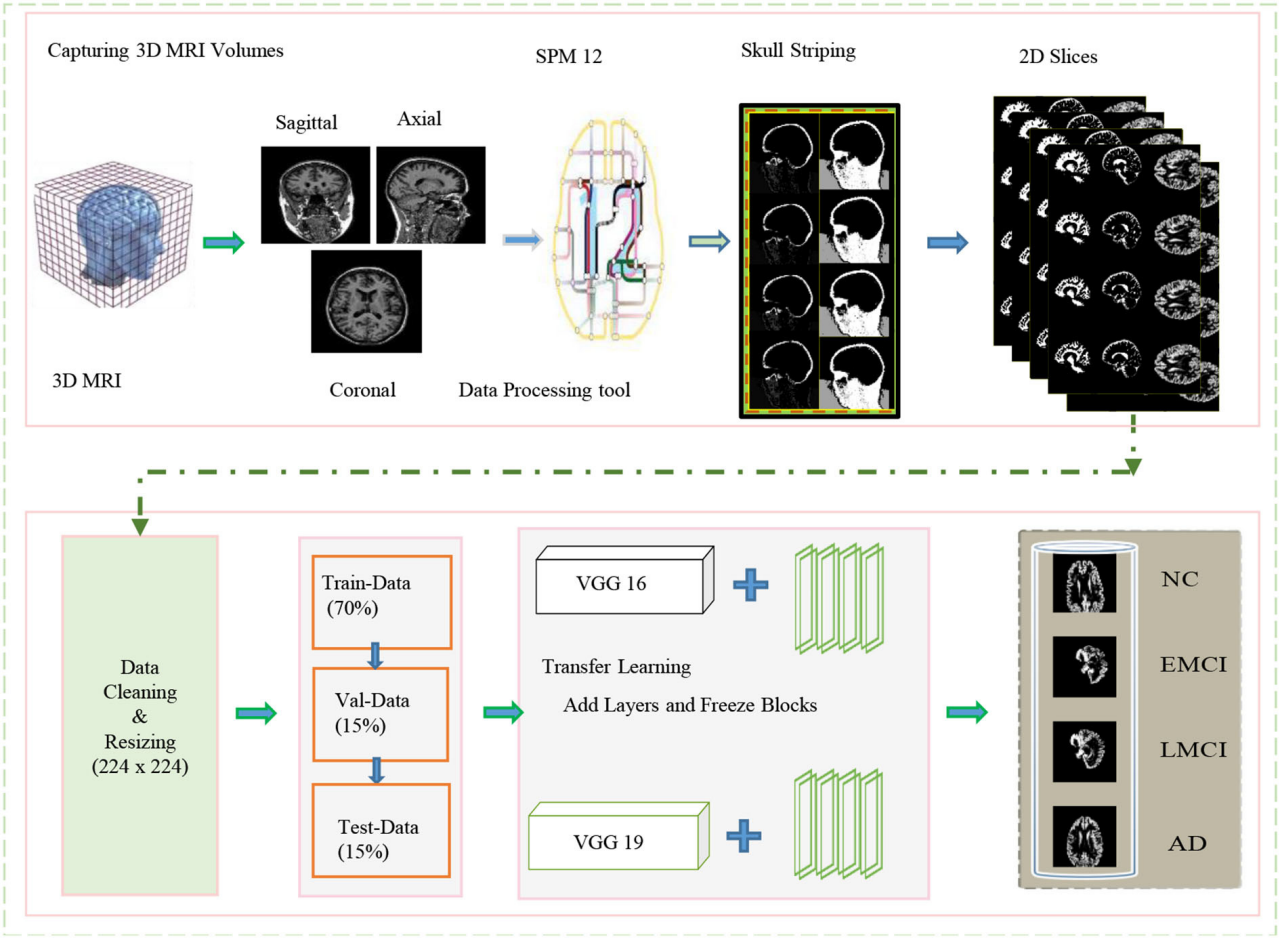
Overview of the proposed framework for processing data and training in the network for classification.

**Figure 2:**
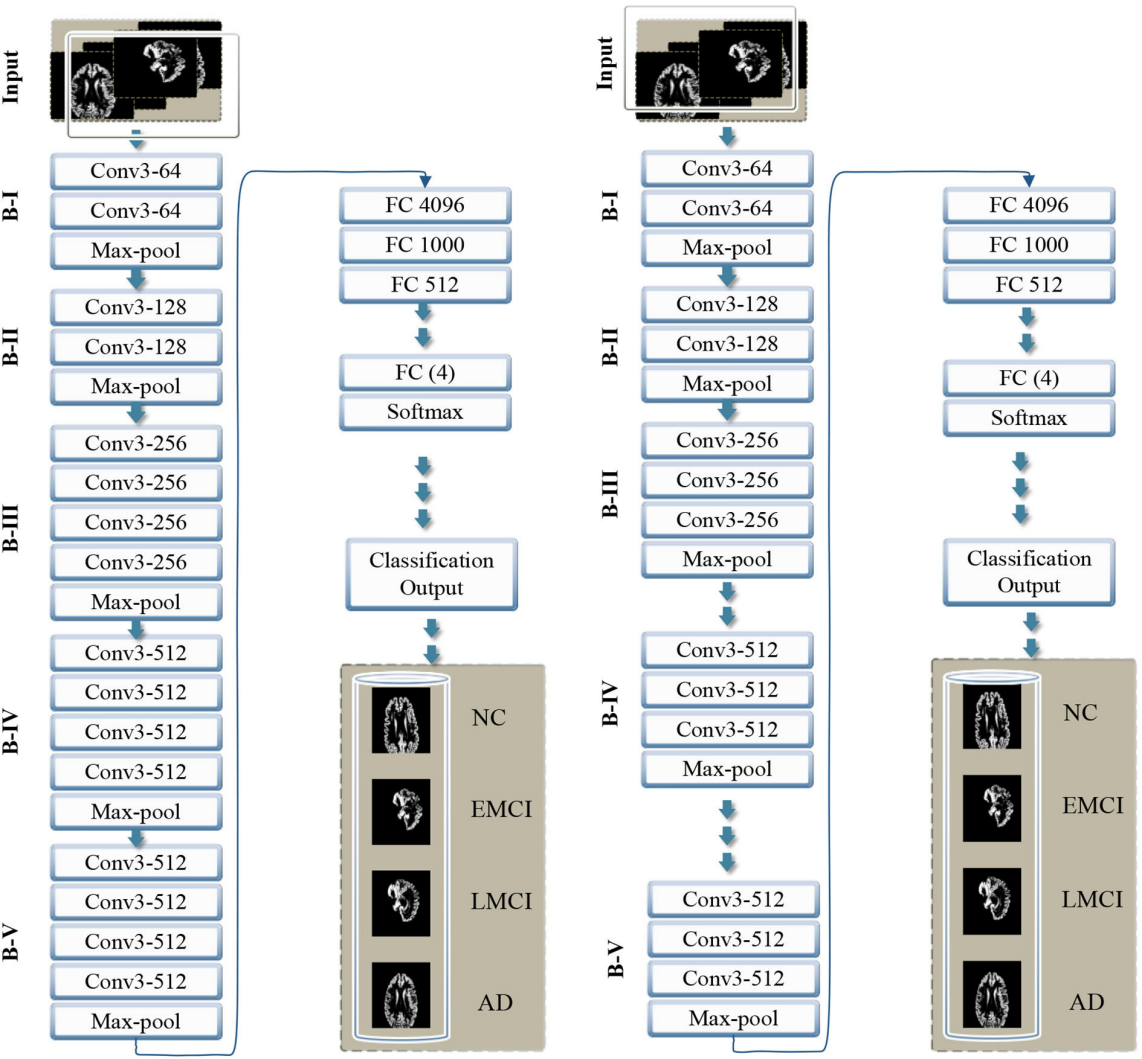
Overview of the proposed framework with step-wise block (B) representation as B-I to B-V. The left side shows VGG 16, and the right side shows the VGG 19 model with the proposed layer-wise transfer learning in both networks, blocks are frozen from B-I to B-V alternatively.

In this study, we proposed a transfer learning-based multi-class classification for the early diagnosis of AD. The patients' data in this study was obtained from the ADNI database. We gathered 315-T1 weighted MRI images of four classes: NC, EMCI, LMCI, and AD. The processing overview of these images is shown in Figure 1. We extracted the gray matter through these 3D voxels and utilize these GM slices to train VGG architectures, as shown in Figure 2. We utilized the weights from the pre-trained network on ImageNet and adopted a layer-wise transfer learning while step-wise freezing the blocks. The proposed framework successfully classifies various stages of AD and yields significant results by using only a small-scale dataset.

### 3.1. Datasets and pre-processing on data

The magnetic resonance imaging technology is continually being improved and developed by researchers, and it has revolutionized the way neurological disorders are discovered and diagnosed. Whereas, the inconsistent intensity scale of the image makes it difficult to visualize and evaluate the data manually (Lundervold and Lundervold, 2019). It is essential to provide the correct information during learning if learning-based technologies are to produce accurate predictions. Thus, data preprocessing is a crucial step to manage to increase the contrast and pixel intensity. We applied several pre-processing operations on the available MRI scans of several patients obtained from the ADNI data repository. The specification of the dataset utilized in this work is shown in Table 1. A different number of subjects are selected to handle the class imbalance problem. We mainly exclude the opening and closing slices of the MRI due to the limited information contained in these slices. Images (i.e., 3D MRI scans) obtained from ADNI are available in neuroimaging informatics technology initiative (NIFTI) format. We pre-process these images by utilizing the statistical parametric mapping tool (SPM12) for the tissue-wise segmentation of the input into gray matter, white matter, and cerebrospinal fluid (CSF). We perform several operations (i.e., skull striping, registration, normalization, and segmentation) to extract the 2D-MRI PNG images from the available MRI scans.

**Table 1:** Sample size of the dataset with specifications and number of subjects utilized for each class.

**Type**	**Subjects**	**Age**	**MMSE**	**Slices**
NC	75	73 ± 8.5	26.5 ± 1.4	4637
EMCI	75	74 ± 7.7	29.5 ± 1.2	1882
LMCI	80	72 ± 7.9	28.5 ± 1.6	4558
AD	85	75 ± 9.5	24.5 ± 1.9	3349

In this study, we focused only on the gray-matter slices to detect the memory loss changes in early AD detection. We consider the ICBM space template for affine regularization and a bias regularization of 1*e*^−^3 with a full bias width at half maximum of 60 mm cutoff. Once these prepossessing operations are complete, we resize all the images to a size of 224 × 224. These images are suitable for training and testing and analogous to the size of ImageNet. The subjects involved in this dataset are scanned with respect to different durations of visits of the patients. Every scan is a different subject and contains the GM, WM, and CSF slices; of which, GM slices are fed to the network to interpret the usable information extracted through MRI volumes. In this regard, we split our dataset into training (70%), testing (15%), and validation data (15%).

## 4. Neural networks and transfer learning framework

Convolutional neural networks perform convolutional operations to extract the features from the input data. These features are learned during the training process. Later on, the network's prediction behavior determines the network's learning quality. In artificial intelligence (AI), CNNs are distinguished from the other type of networks due to the superior performance of these networks in the multiple-image processing and visualization domains. The main type of layers in these networks are convolutional layers. These are the first layers that extract the features which are pooled with pooling layers. Finally, the fully connected layers are applied. Considering the recent advancements in AI and neural networks for feature extraction and associated task, we employed VGG 19 and VGG 16 architecture with a transfer learning approach for the target problem.

Transfer learning is a method for developing a predictive model for a separate but related problem that can then be utilized partially or entirely to speed up training and ultimately enhance the model's performance for the problem. It involves applying a previously trained model to new problems. Transfer learning-based techniques are now very popular in the field of medical image processing. Considering the advantages of automatic feature extraction and identification with the help of a pre-trained model have proved to be very useful in the target domain. Using a pre-trained model saves the time and effort of building a new model from scratch. It is also difficult to train a substantially large-scale network without amassing millions of annotated images. Therefore, using the pre-trained weights of a network with precise tuning on fresh data is a distinct and advantageous approach. Two models adopted in this study for transfer learning are pre-trained on the ImageNet database comprising millions of images.

The existing models, for example, Inception-Net with 23.62 million parameters, Xception-Net with 22.85 million, and ResNet with 23 million parameters, can also be used for transfer learning. However, considering the importance of the target problem, we selected VGG architecture with 138 million parameters. Feature transfer can be problematic in particular circumstances, such as when the datasets are small or imbalanced. Because transfer learning will not be effective if the final classification layer's characteristics are insufficient to distinguish the classes for a particular problem. Thus if the datasets are not comparable, the feature will hardly be transferred. We present transfer learning results in two categories, i.e., VGG 16 and VGG 19. Thus in each case, we propose to freeze some of the blocks in the network while freezing some fully connected layers.

## 5. VGG model with proposed transfer learning and experimental evaluations

The pretrained VGG-16 and VGG-19 models utilized in this study are trained on the ImageNet database. We consider freezing the weights and leveraging a pretrained convolutional base. We also include fully connected classification layers to transform the network for our multi-class classification task. The first layer in the model learn feature extraction. The initial layers learn to extract the generic features, and the final layers learn the target-oriented features. We added the new fully connected dense layers and performed several experiments with the rectified linear unit (ReLU) activation function. The main objective of the activation function is to induce non-linearity in the data. It can be expressed for an input value (v) as below.


(1) $$\begin{array}{l} ReLU\left(v\right)=max\left(0,v\right) \end{array}$$

(2) $$\begin{array}{l} ReLU(x)=\{\begin{array}{c} 0,\text{ if v}<0\\ \text{x},\text{ if v}\geq 0 \end{array} \end{array}$$

The final layers in both of the models (i.e., VGG 16 and VGG 19) use the Softmax function. The number of neurons is reduced to 4 due to four classes (i.e., NC, EMCI, LMCI, and AD) in the target problem. The softmax function is the generalization of logistic regression, which is utilized in the classification of mutually exclusive classes. It converts the values to a normalized probability distribution of input for user display. Therefore, it is utilized at the final layer of the network and expressed as σ for input vector *v*_*i*_ and standard exponential *e* followed by a normalization factor with a summation ∑ at the bottom for the *K* number of classes in the multiclass classifier.


(3) $$\begin{array}{l} \sigma (v\vec{)}i=\frac{e^{v_{i}}}{\sum_{j=1}^{K}e^{v_{j}}} \end{array}$$

The loss function utilized in this regard is categorical cross-entropy loss which exponentially penalizes error in the probability prediction. The target problem involves predictions for more than one class, i.e., NC, EMCI, LMCI, and AD, and involves 4 labels. Therefore, loss function for the network also varies accordingly, and we consider categorical cross-entropy loss for these multiple-class classification problems. The categorical cross-entropy loss with *p*_*i*_ probabilities for *i*^*th*^ labels with truth values *t*_*i*_, for the N number of classes is expressed as $L_{CrE}$.


(4) $$\begin{array}{l} L_{CrE}=\overset{n}{\underset{N=1}{\sum }}\overset{n\left(size\right)}{\underset{i=1}{\sum }}t_{i}log\left(p_{i}\right) \end{array}$$

Our goal is to reduce the loss as much as possible. In each case, the cross-entropy for the *i* number of classes was estimated for the dementia's *d*_*i*_, where *i* = 1....5 in this case. The probability of the output *y* for each class can be estimated for dementia, where the cross-entropy for each category NC, EMCI, LMCI, and AD is *CE*_*NC*_, *CE*_*EMCI*_, *CE*_*LMCI*_, and *CE*_*AD*_, respectively.

### 5.1. Experiments and evaluation metrics

We categorize our experiments based on VGG 16 and VGG 19 models. In each category, we perform several experiments. The overall performance is measured in terms of confusion matrices (Deng et al., 2016) where true positives (TP), true negatives (TN), false positives (FP), and false negatives (FN) give an overview of the accuracy, specificity, sensitivity, and precision. We also measure f1-score in addition to accuracy, specificity, sensitivity, and precision. Each column in the confusion matrix is a visual tool to understand the predicted score, where columns and rows show the true and predicted labels, respectively.

### 5.2. Evaluation metrics

In order to evaluate the performance of the proposed framework, the confusion matrix consists of TP, FP, TN, and FN. Thus, an overview of the confusion matrix gives a comprehensive overview of the F1-score, specificity (Sp), sensitivity (Se), accuracy (Ac), and precision (Pr) value because all of these metrics follow TP, TN, FP, and FN. Witten and Frank (2002). The mathematical expressions below depict this relationship explicitly.

#### 5.2.1. Positive predictive value

The positive predictive value is called the precision and shows the portion of real positive cases.


(5) $$\begin{array}{l} Pr=\frac{TP}{TP+FP} \end{array}$$

#### 5.2.2. Sensitivity

Sensitivity is the recall value that shows the actual positive and the correctly predicted portion of values. This metric reflects correctly anticipated cases and depicts the coverage of real positive cases, also termed the true positive rate (TPR).


(6) $$\begin{array}{l} Se=\frac{TP}{TP+FN} \end{array}$$

#### 5.2.3. Specificity

Specificity is associated with the likelihood of a negative test rate in the absence of the condition and is considered a true negative rate.


(7) $$\begin{array}{l} Sp=\frac{TN}{TN+FP} \end{array}$$

#### 5.2.4. Accuracy

Classification accuracy is a statistical measurement that evaluates the performance of a classification model by dividing the number of correct predictions by the total number of predictions.


(8) $$\begin{array}{l} Ac=\frac{TP+TN}{TP+FN+TN+FP} \end{array}$$

#### 5.2.5. F1 measurement

The F-Metric is a method for combining accuracy and recall into a single measure that encompasses both and is widely utilized in classification tasks.


(9) $$\begin{array}{l} \begin{array}{l} F1-Measure=\frac{TP}{TP+\frac{1}{2}(FP+FN)} \end{array} \end{array}$$

In addition, we also present graphical and numeric results in terms of accuracy for each class.

### 5.3. Experimental settings and results

**Figure 3:**
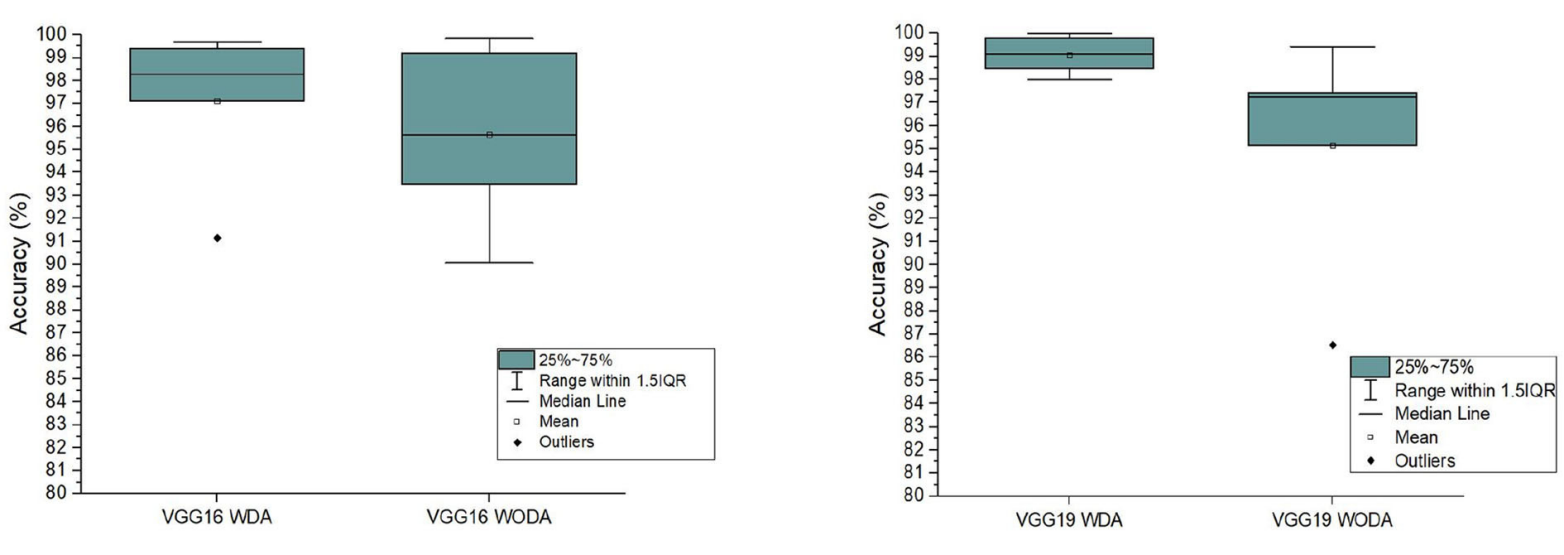
The comparison of the accuracy of the proposed frameworks with data augmentation (WDA) and without data augmentation (WODA).

Experimental results for precision, accuracy, and F1 score of VGG 16 and VGG 19 (with and without data augmentation) are presented in the Table 2. In this table, we show the results for NC, EMCI, LMCI, and AD, respectively. The overall performance of the network with and without data augmentation is also shown in this table. The average accuracy of VGG 16 with and without data augmentation for all four classes is 96.39%. Similarly, the average accuracy of the VGG 19 with and without data augmentation for all four classes is 96.81%. This comparison demonstrates that VGG 19 performs slightly better than VGG 16. We also demonstrate the fact with the help of box plots shown in Figure 3. The results are shown for VGG 16 and VGG 19 with data augmentation (WDA) and without data augmentation (WODA).

**Table 2:** The comparison of the evaluation metrics in terms of precision, accuracy, and F1 score for VGG16 and VGG19 with and without data augmentation.

**Model**	**VGG16 (Without DA)**			**VGG16 (With DA)**		
**Metrics**	**Precision**	**Accuracy**	**F1 Score**	**Precision**	**Accuracy**	**F1 Score**
NC	91.71	99.20	95.30	97.65	99.40	98.51
EMCI	99.60	90.07	93.61	99.22	91.13	95
LMCI	96.06	99.85	97.91	96.46	99.70	96.02
AD	99.38	93.52	96.36	99.56	98.27	98.91
**Model**	**VGG16 (Without DA)**			**VGG16 (With DA)**		
**Metrics**	**Precision**	**Accuracy**	**F1 Score**	**Precision**	**Accuracy**	**F1 Score**
NC	97.65	97.41	96.82	97.27	99.80	98.51
EMCI	99.22	86.52	92.43	100	99.09	99.54
LMCI	96.46	99.41	97.91	98.41	100	99.19
AD	99.56	97.26	98.39	99.85	97.98	98.90

The proposed framework's overall performance is much better compared to the existing methods. To demonstrate the fact we present the comparison of the proposed method with several state-of-the-art approaches. The results of this comparison are shown in Table 3. In addition, we also compare the performance of the proposed framework with other state-of-the-art models, including DenseNet, Inception ResNet V2, AlexNet, Inception V3, ResNet101, and ResNet50. The results for comparison of these methods with the proposed framework are shown in Table 4. This table shows that the proposed framework (i.e., VGG19+ALFB, VGG16+ALFB) outperformed the other models. To demonstrate the performance of our method with respect to the existing state-of-the-art approaches, we provide a comprehensive comparison with several methods. The competitor approaches include a deep sparse multi-task learning for feature selection in the diagnosis of AD (DSMAD-Net) (Suk et al., 2016), CNN-expedited (Wang et al., 2017) classification of AD on imaging modalities (CAIM) (Aderghal et al., 2018), a transfer learning approach for the early diagnosis of AD on MRI image (TLEDA-Net) (Mehmood et al., 2021), multi-domain transfer learning (TL), a matrix similarity-based method (Zhu et al., 2014), multimodal learning (Liu et al., 2014), multimodal stacked Net (Shi et al., 2017), shape-attributes of brain structures as biomarkers for AD (SABS-AD) (Glozman et al., 2017), detecting AD on small dataset (Li et al., 2018), multimodal neuroimaging feature learning with multimodal stacked deep polynomial networks for diagnosis of AD (MMSPN-AD) (Basaia et al., 2019), multimodal classification of AD diagnosis (MMC-AD) (Tong et al., 2017), predicting AD with a 3D neural network (3D-CNN-PAD) (Payan and Montana, 2015), natural image bases to represent neuroimaging data (NIBR-Net) (Gupta et al., 2013), a differential diagnosis strategy (Sørensen et al., 2017), a transfer learning bases method (Naz et al., 2022), a convolutional neural networks-based MRI image analysis for the AD prediction from MCI (Lin et al., 2018), and a novel end-to-end hybrid network for AD detection using 3D CNN and 3D CLSTM (Xia et al., 2020). We performed several experiments, while the freezing pre-trained base and step-wise tested the performance for several blocks in VGG 16 and VGG 19. We include additional layers (ADL) and perform experiments with a block-freezing strategy. We independently present the results of the effects of the proposed changes on VGG16 and VGG19.

**Table 3:** Comparison of the accuracy of the various state-of-the-art approaches with the proposed work.

**References**	**Methods**	**Modalities**	**Distinction**	**Data**	**Accuracy (%)**
Tong et al. (2017)	MMC-AD	MRI, FDG-PET	AD, NC, MCI	ADNI	72.9
Wang et al. (2017)	CNN-Expedited	MRI	NC, MCI	OASIS	90.6
Payan and Montana (2015)	3D-CNN-PAD	MRI	NC, MCI, AD	ADNI	85.3
Gupta et al. (2013)	NIBR-Net	MRI	NC, MCI, AD	ADNI	78.2
Cheng et al. (2017)	Multi-Domain TL	MRI	NC, MCI	ADNI	94.7
Sørensen et al. (2017)	Differential Diagnosis	sMRI+Volumetry HS+CT	MCI & AD	ADNI & AIBL	62.7
Zhu et al. (2014)	Matrix-Similarity	MRI+PET, CSF	MCI converter, MCI-NC, AD	ADNI	72.6
Liu et al. (2014)	Multimodal learning	MRI+PET	AD, NC, MCI	ADNI	53.8
Suk et al. (2016)	DSMAD-Net	MRI+PET	NC, MCI, AD	ADNI	62.9
Shi et al. (2017)	Multimodal Stacked Net	MRI+PET	AD,NC, cMCI/MCI	ADNI	57.0
Glozman et al. (2017)	SABS-AD, Transfer-Learning (ImageNet)	MRI	AD, MCI, NC	ADNI	83.5
Aderghal et al. (2018)	CAIM, Transfer-Learning	MRI	AD, MCI, NC (2-way classification)	ADNI	72.91
Li et al. (2018)	Transfer-Learning	MRI	AD,NC	ADNI	84
Shi et al. (2017)	MMSPN-AD, Transfer-Learning	MRI	AD,NC, MCI	ADNI	75.1
Lin et al. (2018)	CNN	MRI	MCI to AD conversion	ADNI	88.79
Xia et al. (2020)	3D CLSTM, CNN	MRI	AD,NC, MCI	ADNI	94.19
Mehmood et al. (2021)	TLEDA-Net	MRI	AD, MCI, LMCI, NC 2 way classification	ADNI	83.64
Naz et al. (2022)	Transfer learning (Avg on Vgg-19)	MRI	AD, MCI, NC 2 way classification	ADNI	98.12
VGG 19+ALFB (Ours)	Transfer-Learning (VGG16+ALFB)	MRI	NC, EMCI, LMCI, AD (4-way classification)	ADNI	98.47
VGG 16+ALFB (Ours)	Transfer-Learning (VGG16+ALFB)	MRI	NC, EMCI, LMCI, AD (4-way classification)	ADNI	97.12

**Table 4:** Comparison of the proposed architectures with adding layer and freezing blocks (ALFB) in VGG16 and VGG-19, with the existing state-of-the-art architectures in terms of accuracy.

**Networks**	**Accuracy**	**Networks**	**Accuracy**
DenseNet	92.70	Inception V3	88.33
Inception ResNetV2	85.82	ResNet101	91.56
AlexNet	89.33	ResNet50	93.98
VGG-19+ALFB (Ours)	98.47	VGG-16+ALFB (Ours)	97.12

**Figure 4:**
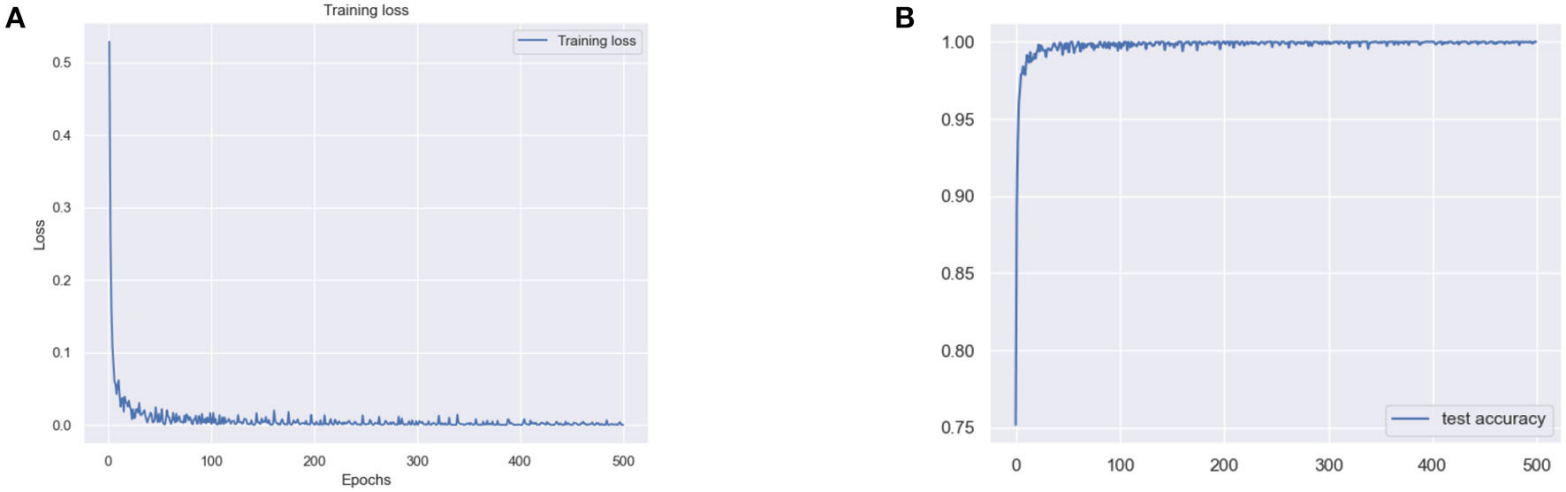
**(A)** Training loss and **(B)** test accuracy of the VGG 16 model with additional layer and freezing the block.

**Figure 5:**
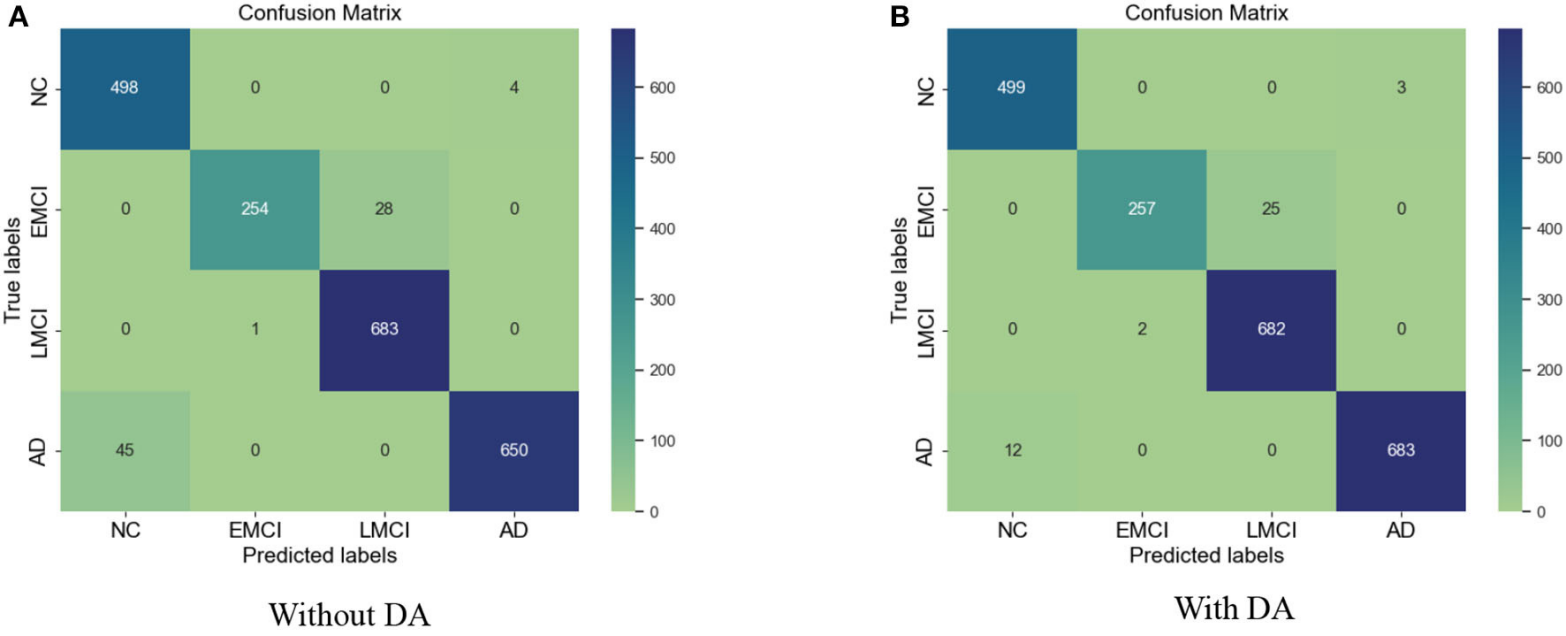
**(A)** Confusion matrix without augmentation. **(B)** Confusion matrix with augmentation for the VGG 16.

The results for the training loss and test accuracy with additional layers and frozen blocks in the VGG16 model is shown in Figure 4. The resulting confusion matrices to evaluate the network's performance in terms of true and predicted labels are shown in Figure 5. The confusion matrices are shown for (Figure 5A) without data augmentation and (Figure 5B) with data augmentation. The comparison of the accuracy is shown in Table 2 for corresponding confusion matrices, the data augmentation slightly improves the network performance.

**Figure 6:**
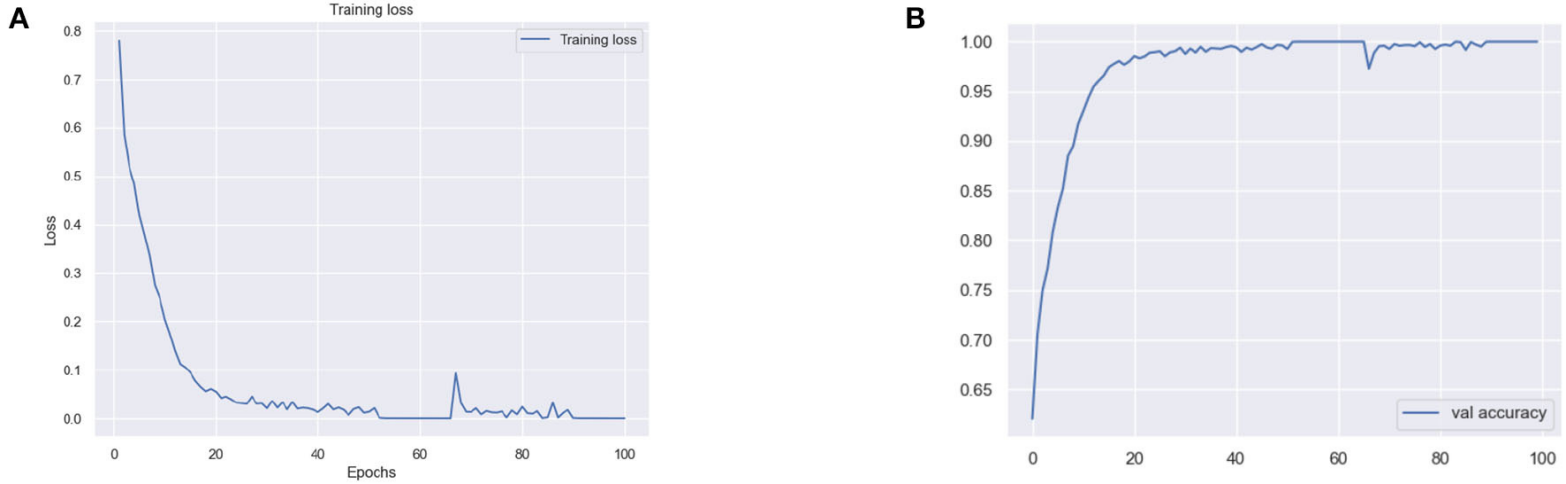
**(A)** Training loss and **(B)** validation (val) accuracy of the VGG 19 model with additional layer and freezing the blocks.

**Figure 7:**
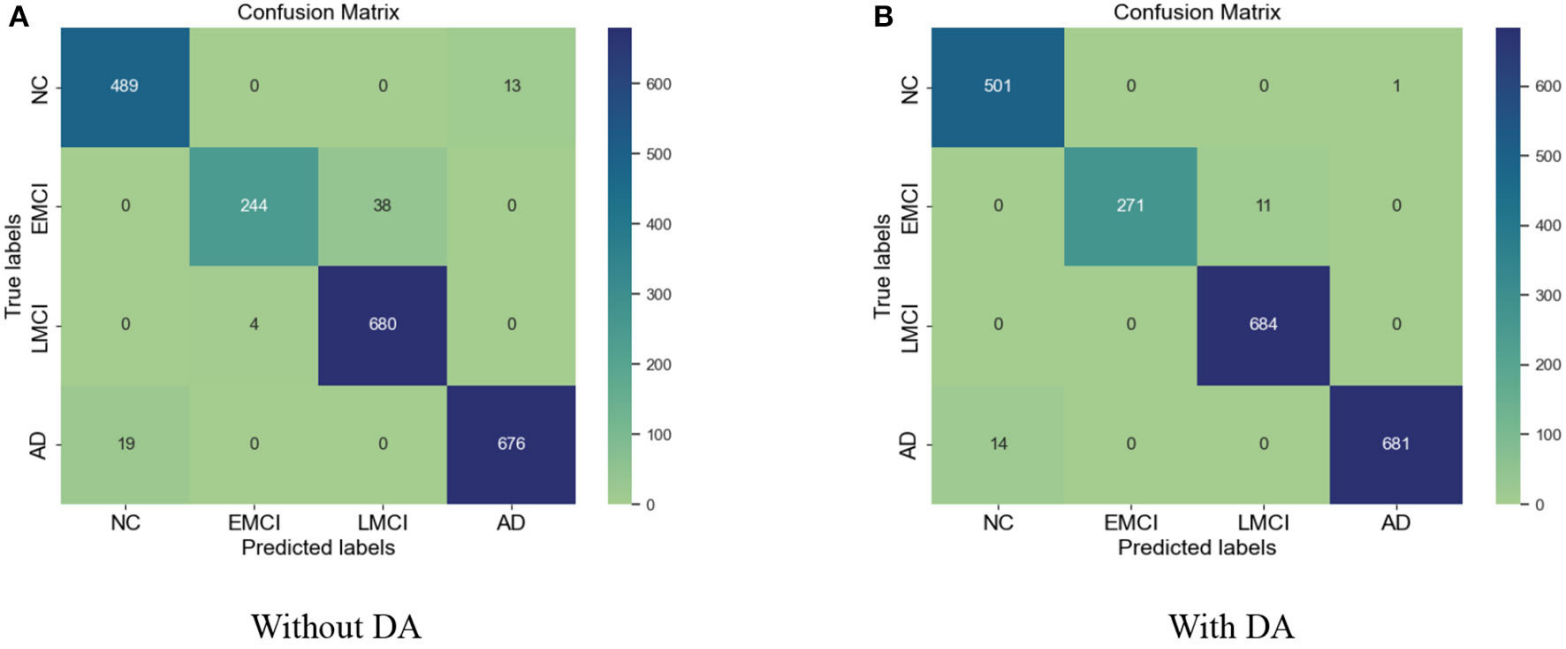
**(A)** Confusion matrix without data augmentation (DA). **(B)** Confusion matrix with DA for VGG 19.

Similarly, for the VGG 19 model, we show the results for the training loss and testing accuracy in Figure 6, whereas the corresponding confusion matrices are shown in Figure 7. The comparison of the confusion matrices and the accuracy in Table 2 demonstrate that VGG 19 performs slightly better than VGG 16. Comparing the confusion matrices for both networks depicts that the data augmentation slightly improves the overall performance of the networks during transfer learning. In Table 2 we present the results for the specificity, sensitivity, and f1-score for VGG-16 and VGG19 with additional layers and freezing blocks. It depicts the performance and overall efficiency of the proposed method. To improve the robustness and fully-fine tune the model, we propose to increase the data size with data augmentation. It proved to be useful because gathering a large-scale dataset in the target domain is a great challenge. In comparison, the network-based methods are heavily dependent on large-scale datasets. The data augmentation improves the data's size significantly and resolves the challenges of class imbalance and over-fitting.

## 6. Conclusion

This study presents a transfer learning-based approach to diagnose various stages of AD. We intend to automate the detection of various stages of Alzheimer's to ease the burden on the healthcare system. We use the pre-trained VGG architectures, i.e., VGG 16 and VGG 19, and propose a layer-wise transfer learning while adding layers and step-wise freezing the blocks in the pre-trained architecture. We leverage the pre-trained convolutional base, fine-tune the model for our 4-way classification on MRI images, and obtain state-of-the-art results in the target domain. The proposed layer-wise transfer learning significantly improves the framework's performance, where we also resolve the challenges of class imbalance and small data samples. The comparison with existing methods demonstrates that the proposed framework is superior in terms of accuracy and prediction. We achieved an accuracy of 97.89% for the proposed 4-way classification.

## Data availability statement

The original contributions presented in the study are included in the article/supplementary material, further inquiries can be directed to the corresponding author.

## Author contributions

RK contributes to the conceptualization, methodology, data curation, and writing of the original draft preparation with software and validation process. All authors also conceived in the process and approved the manuscript for submission.
